# Properties of a neutral, thermally stable and surfactant-tolerant pullulanase from worker termite gut-dwelling *Bacillus safensis* as potential for industrial applications

**DOI:** 10.1016/j.heliyon.2022.e10617

**Published:** 2022-09-13

**Authors:** Oladipo Oladiti Olaniyi, Afolayan Olalekan Damilare, Olusola Tosin Lawal, Festus Omotere Igbe

**Affiliations:** a Department of Microbiology, Federal University of Technology, PMB 704, Akure, Nigeria; b Department of Biochemistry, Federal University of Technology, PMB 704, Akure, Nigeria

**Keywords:** Termites' gut, Pullulanase, Purification, Biochemical properties, Surfactants

## Abstract

The gut of termite has been observed to host communities of bacteria which exhibited pullulan-degrading ability. *Bacillus safensis* displayed maximum pullulanase (a debranching enzyme) activity and it was therefore selected for production, purification and characterization of pullulanase which was the aim of the study. The crude enzyme obtained from the pullulanase production medium was subjected to ammonium sulphate precipitation, ion exchange and gel-filtration chromatography and the physicochemical properties of the purified was thereafter characterized. A purified pullulanase with the yield of 13% and 24-fold purification was obtained and its homogeneity was established by molecular weight of 42 kDa. The optimum pH 7 and 60 °C were obtained while the enzyme was stable between 40-60 °C and pH 4–5 and 7–8 respectively with significant amount of residual activities recorded. The purified pullulanase was stimulated in the presence of Ca^2+^, urea and SDS while Al^3+^, Fe^2+^, Co^2+^, Cu^2+^, Mg^2+^ and chelating agent, EDTA mildly inhibited the activity of the enzyme in a concentration-dependent manner. The *K*_*m*_ and *V*_*max*_ were found to be 0.324 μmol/ml/min and 6.85 mg/ml respectively. The exceptional physicochemical properties of *B. safensis* pullulanase could find application in several industrial processes.

## 1 Introduction

Pullulan, an intermediate structure between amylose and dextran is produced by *Auerobasidium pullulans*, a polymorphic fungus (Shukla et al., 2019). Pullulan is made up of α-1,4 and α-1,6 glycosidic linkage which offers the structure restriction against desiccation and predation (Farris et al., 2014; Ogbo and Nwozor, 2020). Breaking down of pullulan requires specific enzyme, as common amylolytic enzymes such as α-amylase and β-amylase could not hydrolyse the compound due to its complexity (Zebardast et al., 2017). In line for various industrial and commercial applications of pullulan, its degradation and enzymatic conversion is highly required, so that variety of useful products could be obtained. However, this complex structure could only be broken down by specific glucanase amylolytic enzyme called pullulanase, a debranching enzyme (Da Cruz, 2013; Zhang et al., 2020).

Debranching enzymes are capable of catalyzing α-1,6 glycosidic linkages thereby leading to their hydrolysis (Wu, 2015; Dakhmouche et al., 2021). Debranching enzymes are classified into indirect and direct branching enzymes. Amylo-1,6 glucosidases, indirect debranching enzymes produced by animals and yeast are only capable of hydrolysing a 1,6 - α branch point while pullulanases and isoamylases, classes of direct debranching enzymes found in plants and bacteria have been observed to effectively break α-1,6 glucosidic bond of unmodified substrate (Hii et al., 2012; Zhang et al., 2020). Therefore, pullulanase (pullulan α-glucano hydrolase; EC 3.2.1.41) has been highly preferred in industrial processes because of its capability to be involved in saccharification of starch, amylopectin, pullulan and related polysaccharides in combination with other amylolytic enzymes and subsequently yield hydrolysis of α-1, 6 bonds in the polysaccharides with the production of maltose, maltotriose and fructose (Wu et al., 2015; Ma et al., 2015; Dakhmouche et al., 2021). More so, its usefulness as additives, production of malto syrups, pure glucose and fructose and reduction of dental plaque has drawn more attention of its relevance and requirement (Zebardast et al., 2017; Dakhmouche et al., 2021).

Pullulanases are widely classified according to their substrate specificities and reaction products (Ma et al., 2015). Most pullulanases that are found in different species of bacteria, yeast, and fungi have been reported to be type II pullulanases (Kim et al., 1993; Dakhmouche et al., 2021) while few numbers of type *I pullulanases* that have been observed are found at gene level (Suzuki et al., 1991). It has however been observed also that, Type I and type II pullulanases work synergistically: Type I acts on linkages in pullan and amylopectin, a branched oligosaccharide yielding maltotriose and oligosaccharides that are branched while Type II pullulanase which has amylase and pullulanase activity releases remaining polysaccharides by cleaving starch glycosidic bonds at α-1,6 and α-1,4 glycosidic linkage (Zebardast et al., 2017). A thermostable and thermo-tolerant enzyme such as pullulanase is desirable in the course of starch bioprocessing because the process requires solubility, low risk microbial contamination and viscosity, decreased reaction periods which could only be achieved using an enzyme with these properties (Zebardast et al., 2017; Iqrar et al., 2020). Hence, there is constant search for sources of pullulanase-producing microorganisms especially bacteria with novel physicochemical properties such as thermostability, pH-stable, metal ions and inhibitor tolerant as the starch-processing industries are increasing.

Termite, a macro-invertebrate is a dominant animal in its ecosystem with the ability to feed on dead organic materials such as dead log of woods and it is therefore referred to as decomposer (Vargo, 2019). Termite is in mutual relationship with the dead woods (Ulyshen, 2016), that is; it is provided with the battery of microorganisms which enable it to breakdown complex compounds such as cellulose, starch, lignin and even pullullan that are present in the dead woods (Ashton et al., 2019) while the enzymes released by the microbes could in turn hydrolyse the structures responsible for hardening of wood. These communities of microorganisms (bacteria and fungi) have become inherent part of termite and then making it possible for the animal to degrade any carbohydrate-related macromolecule. Since, pullulan is a complex compound that requires special class of enzyme for its degradation, bacteria inhabiting gut of termites have been observed to secret pullulan-degrading enzyme with novel physicochemical properties that are desirable in several industrial processes.

However, pullulanase has been reported from bacterial origin such as *Bacillus lactis* (Wasko et al., 2011), *B. halodurans* (Asha et al., 2013), *B. cereus* (Waleed et al., 2015) and *Paenibacillus polymyxa* Nws-pp2 (Ma et al., 2015); meanwhile in this study *Bacillus safensis* isolated from the gut of termite exhibited maximum pullulanase-producing ability of all the bacterial isolates screened for pullulanase production and it was further investigated for its exceptional biochemical properties which could be useful in industrial and commercial applications.

Hence, the study sought to produce, purify and investigate biochemical properties of pullulanse from *B. safensis* isolated from the gut of termite.

## 2 Materials and methods

### 2.1 Collection and preparation of sample

Worker termites were collected in the morning during raining season between 6 am and 9 am from decaying wood logs within the Federal University of Technology Akure, Nigeria at Obanla area of the campus into a perforated bottle that allowed ventilation. The termites were immobilized on ice for 30 min and then submerged in 70% ethanol for 1 min in order to be surface sterilized. They were thereafter dissected to obtain the guts which were later mashed in a sterile pestle with sterile mortal.

### 2.2 Isolation and purification of bacteria

Exactly 2.50 g of the mashed insect guts were dissolved in 7.5 mL of distilled water in a test tube while a serial of 10^-3^ to 10^-5^ was done with the stock in an aseptic condition. The pour plate method described by Olutiola et al. (2000) was adopted. A 100 μL of the dilution was transferred into a sterile Luria Bertani (LB) agar inside the Petri dish in aseptic environment and was incubated at 37 °C for 48 h. Thereafter, the potential isolates were sub-cultured to obtain pure isolates. The pure isolates obtained were transferred on agar slant of LB agar and kept at −4 °C for further use.

### 2.3 Molecular identification of bacterial isolates

The identities of the best three pullulanase-producing bacteria were authenticated by 16S rRNA genes sequence analysis following standard molecular protocols. The DNA in the isolates was extracted using DNA isolation kit meant for prokaryotes following the manufacturer's prescription. The extracted 16S rRNA genes were amplified in a PCR machine using universals primers (F27: 5′- AGAGTTTGATCCTGGCTCAG-3′ and R1492: 5′TACGGTTACCTTGTTACGACTT3′) following standard PCR protocol. The purity of the amplicons (PCR products) was checked by comparing the bands of the amplicons with the DNA ladder on the agarose gel. After that, the rRNA in the 16S of the small subunit of the ribosome was sequenced and compared with other 16S rRNA nucleotide sequences available in the GenBank using the BLASTN program (http://blast.ncbi.nlm.nih.gov/Blast.cgi) and aligned to their closest relatives by CLUSTX program (Lane, 1991).

### 2.4 Screening of bacterial isolates for pullulanase production

Pullulanase production was carried out using submerged state fermentation according to the method of Nagar et al. (2012). 50 ml mineral salt medium containing the appropriate amount of pullulan (substrate) was prepared in 250 ml Erlenmeyer flasks and autoclaved at 121 °C for 15 min while 25 μl of the inoculum previously prepared in LB broth was inoculated into the sterile culture flasks. The flasks were incubated at 37 °C for 24 h in a rotary shaker at 120 rpm. After incubation, the culture broth was centrifuged at 8100 g for 15 min at 4 °C and the supernatant which was used as source of crude enzyme was collected. The crude enzyme solution was used for the determination of enzyme activities and protein concentration.

### 2.5 Enzyme assay

The pullulanolytic activity was determined using pullulan as the substrate. The reaction mixture contained 0.5 ml of 1% substrate prepared in potassium phosphate buffer pH 6.8 and 0.5 ml of enzyme solution. The control tube contained the same amount of substrate with sterile distilled water in place of crude enzyme. The reaction mixture was incubated at 45 °C for 30 min in water bath. After incubation, the tubes were removed from the water bath and the reaction was terminated by the addition of 1 ml of 3, 5- dinitrosalicylic acid (DNSA) reagent. The tubes were incubated in boiling water bath for 10 min for colour development and cooled rapidly. The activity of the reaction mixture was measured against a reagent blank at 540 nm. One international unit of enzyme activity was defined as the amount of enzyme required to liberate 1 μmol of reducing sugar per minute under standard assay conditions. The specific activity was determined as the number of units of enzyme activity per milligram of enzyme protein (Arotupin et al., 2014; Rahmawati et al., 2016).

### 2.6 Protein determination

Protein concentration was determined using Bradford method (1976) and Nar et al. (2013). 50 μL of the sample (aliquot enzyme) was added to 750 μL of distilled water in the test tube followed by the addition 200 μL of Bradford reagent. The reaction mixture was incubated at room temperature for 10 min. The absorbance was measured at 595 nm using spectrophotometer while the concentration of the protein was extrapolated from the standard curve using serum bovine albumin (BSA).

### 2.7 Optimization of incubation time for pullulanase production

The optimum production of pullulanase was determined by carrying out enzyme production for the period of 48 h while fraction of the culture medium was collected at 6 h interval and pullulanase activity was thereafter measured using standard assay procedure.

## 3 Purification of pullulanase

### 3.1 Ammonium sulphate precipitation

The crude enzyme obtained was precipitated by stepwise addition of calculated amount of solid ammonium sulphate obtained through encorbio.com at 4 °C. The precipitant was left overnight inside refrigerator and was thereafter centrifuged at 22,500 × g for 10 min at 4 °C. The resultant pellet obtained was dissolved in 100 mM potassium phosphate buffer (pH 6.8) and dialysed extensively against the same buffer using Por- 3 R C dialysis membrane tubing (Molecular weight cut-off 3500 Da). The process was maintained at 4 °C.

### 3.2 Ion-exchange and gel filtration chromatography

The dialysate was first loaded onto a DEAE-Sephadex A-50 column (2.5 × 40 cm) previously equilibrated with 100 mM potassium phosphate buffer (pH 6.8) and washed with the same buffer at a flow rate of 60 mL/h. Fractions of 5 mL were collected per tube and the bound protein fractions were eluted with linear gradient of sodium chloride (0–1 M). The presence of protein in the eluted fractions was monitored by measuring their absorbance at 280 nm using UV spectrophotometer (Shimadzu, UV, 1800) and pullulanase activity of the fractions determined using standard assay procedure. The tubes having pullulanase activity were pooled and concentrated by an ultra-filtration system and loaded on Sephadex G-100 gel filtration column (2.5 × 75 cm, flow rate of 20 mL/h) already equilibrated with phosphate buffer (50 mM, pH 6.8). Elution fractions were collected in 5 mL tubes, monitored at 280 nm for the presence of proteins and assayed for pullulanase activity using the standard assay method. Active fractions were pooled and concentrated by ultra-filtration while molecular determination and biochemical characterization were carried on the concentrated purified enzyme to ascertain homogeneity of the enzyme and its physicochemical properties.

### 3.3 Molecular weight determination

The subunit molecular weight of the purified pullulanase and its purity was determined by sodium dodecyl sulphate polyacrylamide gel electrophoresis (SDS-PAGE). This was performed on a 12% polyacrylamide gel using Tris-Glycine-SDS buffer system according to the method of Laemmli (1970). Electrophoresis was performed at 80 V for 4 h using Bio-Rad electrophoresis system (Bio-Rad, UK). The gel was stained with Coomassie brilliant blue R-250 and excess stains were removed by soaking repeatedly with de-staining solution. The visible protein bands of the enzyme and standard protein marker (Bio-Rad prestained molecular weight marker) were observed thereafter.

## 4 Characterization of purified enzyme

The purified enzyme was characterized and their properties were studied. The characters investigated include effect of temperature, pH and metal ion on the activities of the fully purified enzyme (Arotupin et al., 2014).

### 4.1 Effect of pH on purified pullulanase activity

The purified enzyme was incubated at various pH ranging from 3.0 to 11.0. The various buffer systems used were at concentration of 50 mM and includes; glycine-HCl (pH 3.0), sodium acetate (pH 4.0 and 5.0), phosphate buffer (pH 6.0 and 7.0), Tris-HCl (pH 8.0 and 9.0), glycine NaOH (pH 10.0 and 11.0). Each of these buffer solutions was used to prepare 1% pullulan solution used as the substrate in assaying pullulanase activity. The assay was carried out using standard assay procedure.

### 4.2 pH stability of purified pullulanase

The pH stability of the purified pullulanase was determined by preparing aliquot enzyme in different buffers with pH ranging from 4.0 to 9.0: 50 mM and includes; glycine-HCl (pH 3.0), sodium acetate (pH 4.0 and 5.0), phosphate buffer (pH 6.0 and 7.0), Tris-HCl (pH 8.0 and 9.0) and incubated at room temperature for 3 h. Samples were withdrawn at first after 0 min and then at 30 min interval, while the enzyme activity was determined using standard assay procedure.

### 4.3 Effect of temperature on purified pullulanase activity

The activities of purified pullulanase were determined by incubating the reaction mixture containing purified enzyme solution and substrate prepared in potassium phosphate buffer, pH 6.8 at different temperatures (30–90 °C at 10 °C interval) for 90 min. The assay was carried out using standard assay procedure.

### 4.4 Thermostability of purified pullulanase

The thermostability of the purified enzyme was determined by incubating the purified enzymes at different temperatures (40–80 °C at 10 °C) for 1 h. The samples were at first withdrawn at 0 min and subsequently at 30 min interval while the enzyme activity was determined using standard assay conditions.

### 4.5 Effect of metal ions and some chemical agents on purified pullulanase

The effect of various metal ions such as Al^3+^, Na^+^, K^+^, Pb^2+^, Co^2+^, Cu^2+^, Mg^2+^, Mn^2+^ and some chemical inhibitors such as ethylene diamine tetra acetic acid (EDTA), sodium dodecyl sulphate (SDS) and urea were investigated on the activity of purified pullulanase. Concentrations such as 1, 2 and 5 mM of these metal ions and some chemical inhibitors were prepared in potassium phosphate buffer pH 6.8. Equal proportion of substrate metal ion/inhibitor and substrate were incubated with enzyme solution while the enzyme activity was determined according to standard assay procedure (Caglayan et al., 2019).

### 4.6 Determination of kinetic parameters

The kinetic parameters (V_max_ and K_m_) of the purified enzyme were determined by varying the concentration of the substrate (pullulan) from 0 to 1% in 50 mM potassium phosphate buffer (pH 6.8). The enzyme activity was determined according to the standard assay procedure. The apparent kinetic parameters were determined from double reciprocal plots (Lineweaver and Jansen, 1951).

## 5 Results

### 5.1 Screening of bacterial isolates for pullulanase production

Table 1 shows the specific enzyme activities and protein concentrations of all the 26 bacterial isolates. The isolates showed varied specific pullulanase activities which ranged from 0.003 to 0.360 μmol/min/mg. Isolate represented with P4 had the lowest specific pullulanase activity of 0.003 μmol/min/mg, while isolate P1 had the highest activity of 0.360 μmol/min/mg.

**Table 1:** Quantitative screening of bacterial isolates for pullulanase production.

Isolate code	Pullulanase activity (μmol/min/ml)	Protein concentration (mg/ml)	Specific pullulanase activity (μmol/min/mg)
P15	0.116	8.362	0.014
P25	0.16	5.328	0.03
P4	0.022	6.931	0.003
P23	0.151	7.5	0.02
P17	0.267	5.759	0.046
P19	0.041	4.448	0.009
P8	0.236	8.793	0.027
P21	0.099	3.966	0.025
P7	0.247	5.914	0.042
P6	0.118	2.897	0.041
P9	0.346	0.62	0.058
P16	0.1	1.138	0.088
P17	0.082	1.19	0.069
P11	0.516	1.534	0.336
P3	0.06	1.414	0.042
P1	0.484	1.345	0.360
P20	0.174	5.31	0.033
P13	0.254	5.276	0.048
P5	0.078	5.293	0.015
P26	0.105	3.931	0.027
P12	0.462	2.897	0.159
P2	0.174	3.379	0.051
P20	0.077	2.379	0.032
P10	0.116	4.897	0.024
P18	0.14	5.138	0.027

### 5.2 Molecular identities of three bacterial isolates with highest specific enzyme activity

Table 2 reveals the identities of the three selected bacterial isolates with the best pullulanase activities. The sequence obtained was analyzed with BLAST in National Centre for Biotechnology Information (NCBI) database by comparing the genes with those already available in the GenBank. Based on the 16S rRNA sequences, the isolate P12, P1 and P11 were identified as *Bacillus cereus, B. safensis* and *Psychrobacter pulmonis.*

**Table 2:** Molecular identities of the best three pullulanase producing bacteria.

Code	Strain of closest match from NCBI	Maximum identity (%)	16S rRNA Sequence identities
P12	*Bacillus cereus*	94.38	*Bacillus cereus*
P1	*Bacillus safensis*	92.39	*B. safensis*
P11	*Psychrobacter pulmonis*	95.71	*Psychrobacter pulmonis*

### 5.3 Effect of incubation time on the production of pullulanase by the best three isolates

**Figure 1:**
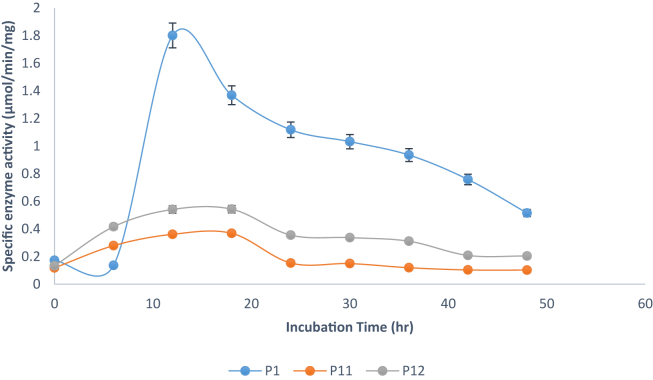
Effect of incubation time on specific pullulanase activities of *B. safensis*, *Psychrobacter pulmonis*, and *B. cereus*. P1 = *B. safensis*, P11 = *Psychrobacter pulmonis*, P12 = *B. cereus*.

Figure 1 show the effect of different incubation periods on pullulanase activity of the best three isolates. *Bacillus safensis* (isolate P1) showed maximum pullulanase production after 12 h with about 1.8 U/mg followed by decrease in enzyme production as incubation hours progressed. *Bacillus cereus* (isolate P12) and *Psychrobacter pulmonis* (isolate P11) exhibited highest pullulanase-producing ability at 18 h with specific activities of 0.5 and 0.35 U/mg, respectively followed by their respective decline in enzyme production as incubation hour increased. Hence, *B. safensis* (isolate P1) was further selected for mass production of pullulanase based on the fact that it had highest specific activity (U/mg).

### 5.4 Elution profile of crude pullulanase from *Bacillus safensis*

**Figure 2:**
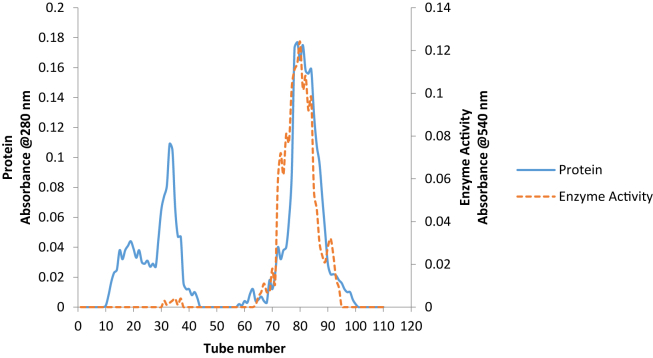
Elution profile of purified pullulanase from *B. safensis* on DEAE Sephadex A-50.

**Figure 3:**
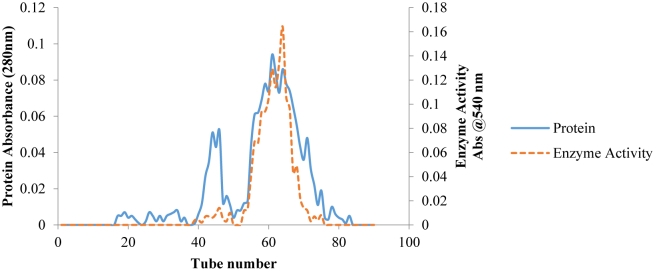
Elution profile of purified pullulanase from *B. safensis* on DEAE Sephadex G-100 Column chromatography.

The summary of purification process is presented in Table 3. The dialysate obtained after dialysis of precipitated protein using ammonium sulphate showed yield of 17% with 6-fold purification. When the dialysate was loaded on DEAE sephadex (ion exchange chromatography), a single sharp peak activity (Figure 2) was observed with 14% recovery and 13-fold purification, while a separation of the pooled eluant from ion exchange chromatography on Sephadex G-100 (Figure 3) finally gave a 13% recovery and 24-fold purification.

**Table 3:** Summary of purification of *B. cereus* extracellular pullulanase

Fractions	Volume (mL)	Enzyme Activity (U/mL)	Protein Conc. (mg/mL)	Total Activity (U)	Total Protein (mg)	Specific. Activity (U/mg)	%Yield	Purification Fold
Crude	850	351.79	0.60	299018	512.38	583.59	100	1
Ammonium Sulfate Precipitation (60%)	116	449.96	0.12	52195	14.08	3707.87	17	6
Ion Exchange Chromatography	78	554.95	0.07	43286	5.53	7826.92	14	13
Gel Filtration	64	604.04	0.04	38658	2.79	13843.75	13	24

### 5.5 Subunit molecular weight of purified pullulanase

**Figure 4:**
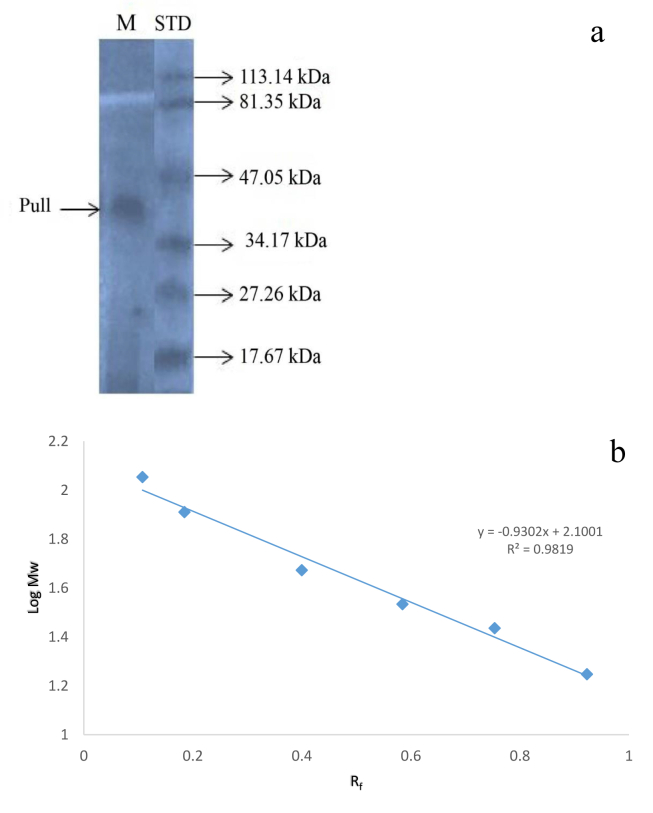
Molecular weight of pullulanase from *B. safensis*. a. Electrophoregram (by sodium dodecyl sulfate polyacrylamide gel electrophoresis (SDS-PAGE)) showing one band molecular weight of purified pullulan of *B. safensis* isolated from the gut of soldier termite. Lane M: purified pullulanase; Lane SD: Standard protein marker. b. Standard graph represents plot of log molecular weight (Mw) against relative mobility (R_f_) of the standard. The molecular of the purified pullulanase was obtained from the equation y = mx + c generated from the graph. The molecular weight was calculated to be 42 kDa.

The determination of molecular weight of purified pullulanase by sodium dodecyl sulfate polyacrylamide gel electrophoresis (SDS-PAGE) and the standard graph of log of molecular weight against R_f_ are presented in Figure 4a and b. The molecular weight of purified pullulanase was obtained from standard curve of Logarithm of molecular weight (M_w_) against R_f_ was estimated to be ≈42 kDa.

### 5.6 Effect of temperature on the activity of purified pullulanase

**Figure 5:**
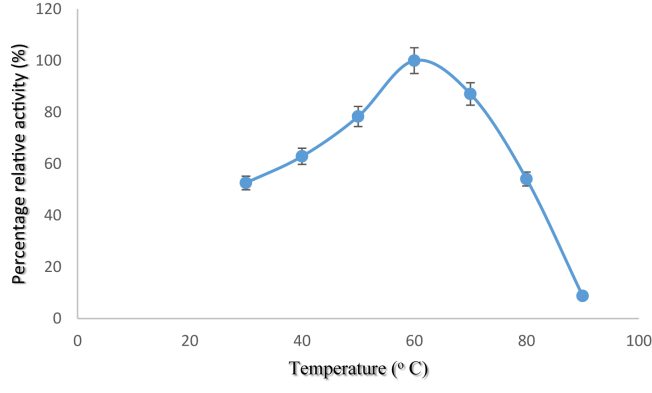
Effect of temperature on purified pullulanase activity from *B. safensis*. The data were collected in triplicate and presented as mean ± standard deviation.

Effect of temperature on pullulanase activity is shown Figure 5. Optimum temperature was observed at 60 °C, while high relative activities of 78 and 87% were observed at 50 and 70 °C, respectively followed by drastic decrease in enzyme activity.

### 5.7 Effect of pH on the activity of purified pullulanase

**Figure 6:**
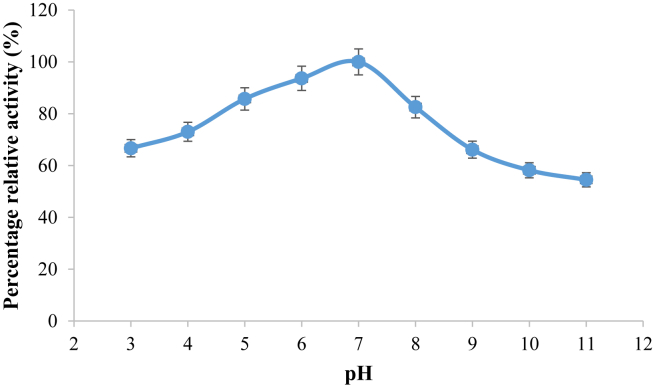
Effect of pH on purified pullulanase activity from *B. safensis*. The data were collected in triplicate and presented as mean ± standard deviation.

The effect of pH on pullulanase activity is shown in Figure 6. Purified pullulanase was optimum at pH 7; meanwhile at pH 5 and 6, very high relative activities of 91 and 94%, respectively were obtained. However, the enzyme was observed to be active at all pH investigated with the least relative activity of 54% recorded at pH 11.

### 5.8 Effect of temperature on the stability of purified pullulanase

**Figure 7:**
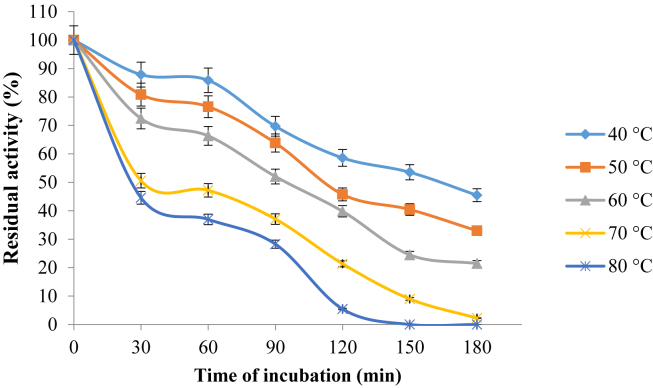
Effect of temperature on the stability of purified pullulanase from *B. safensis*. The data were collected in triplicate and presented as mean ± standard deviation.

The effect of temperature on the stability of purified pullulanase is presented in Figure 7. The purified pullulanase is observed to be most stable at 40 °C with a residual activity of 86%, while 76 and 66% were also obtained at 50 and 60 °C, respectively after 1 h incubation. However, at 70 and 80 °C, the lost its stability but still retained significant residual activities of 47 and 37% respectively after 1 h incubation.

### 5.9 Effect of pH on the stability of purified pullulanase

**Figure 8:**
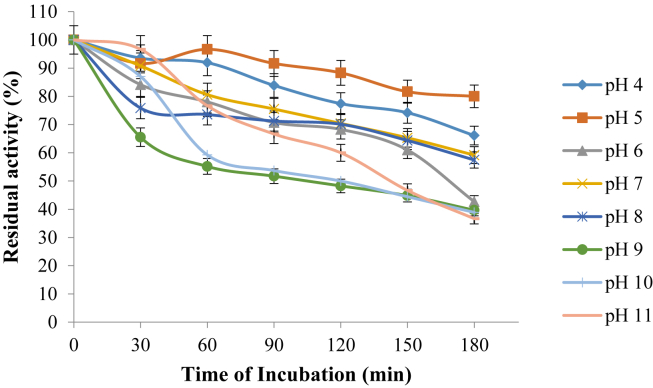
Effect of pH on the stability of purified pullulanase from *B. safensis*. The data were collected in triplicate and presented as mean ± standard deviation.

The effect of pH on the stability of purified pullulanase is presented in Figure 8. The purified pullulanase exhibited maximum stability at pH 5 with 80% of its initial activity recorded after 3 h incubation, while at pH 4, and 7–8, residual activities of 66 and 57% respectively were recorded but the enzyme showed its instability at pH 9–11 with the remaining activity of 37% recorded at 3 h incubation.

### 5.10 Kinetic parameters of the purified pullulanase

**Figure 9:**
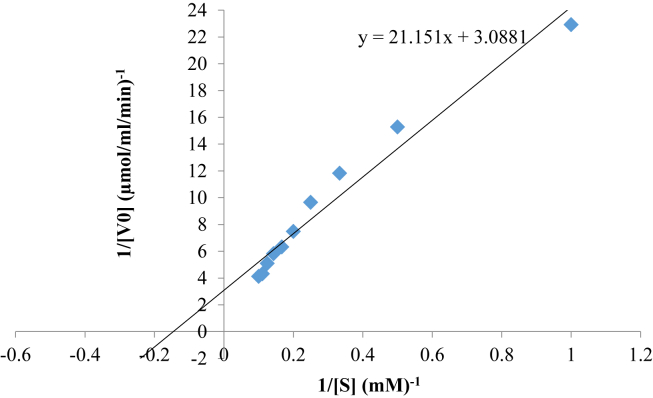
Lineweaver-Burk double reciprocal plot of reaction velocity against substrate concentration for purified pullulanase.

Kinetic parameters of the purified pullulanase is presented in Figure 9. The V_max_ and K_m_ were estimated to be 0.324 μmol/ml/min and 6.85 mg/ml, respectively using pullulan as a substrate.

### 5.11 Effect of metal ions and inhibitors on purified pullulanase

The effect of metal ions and inhibitors is presented in Table 4. The pullulanase activity was stimulated in the presence of Ca^2+^ and urea at all concentrations investigated in a concentration dependent manner, while SDS only activated the enzyme activity at 1 mM concentration. Other metal ions such as Al^3+^, Ca^2+^, Fe^2+^, Pb^2+^, Co^2+^, Cu^2+^, Mg^2+^ and inhibitor such as EDTA mildly inhibited the enzyme activity in a concentration dependent manner.

**Table 4:** Effect of different metal ions and chemical agents concentrations on purified pullulanase activity.

Metal ion	Concentrations (mM)	Pullulanase activity (%)
Control (No metal ion)	-	100
Al^3+^	1	49 ± 0.03
	2	35 ± 0.13
	5	22 ± 0.12
Ca^2+^	1	124 ± 1.01
	2	132 ± 0.21
	5	145 ± 0.11
Fe^3+^	1	47 ± 1.03
	2	36 ± 0.09
	5	23 ± 0.06
Pb^2+^	1	56 ± 0.16
	2	43 ± 0.13
	5	25 ± 0.17
Co^2+^	1	64 ± 0.23
	2	49 ± 0.29
	5	28 ± 0.01
Cu^2+^	1	20 ± 0.13
	2	13 ± 0.21
	5	00 ± 0.00
Mg^2+^	1	62 ± 0.11
	2	49 ± 0.22
	5	11 ± 0.14
EDTA	1	47 ± 1.03
	2	43 ± 0.12
	5	41 ± 0.13
SDS	1	135 ± 0.01
	2	93 ± 0.19
	5	75 ± 0.05
Urea	1	155 ± 0.12
	2	153 ± 0.05
	5	137 ± 0.02

## 6 Discussion

Microbial communities that are capable of breaking down various macromolecules are associated with the gastro-intestinal tract (GIT) of animals (Ray et al., 2012; De et al., 2012; Fajingbesi et al., 2018; Sanni et al., 2019). These microbes are inherently provided with the systems (hydrolytic enzymes) that enable degradation of these macromolecules for their efficient and effective use for growth and development of the animals (Sanni et al., 2019). Of course, hydrolytic enzymes such as phytase, amylase and cellulase are secreted extracellularly, therefore, animals that feed on macro-molecules such as phytic acid, cellulose, pullulan and so on are often supplemented by battery of microbes in their guts for hydrolysis of these complex compounds. Recent studies have shown how microbiota in the gut of fishes have helped in the digestion of foods through the release of various enzyme-degrading biomolecules (Ray et al. 2012; De et al., 2012; Fajingbesi et al., 2018), while Sanni et al. (2019) revealed that gastro-intestinal tract of African giant snail harbors diversities of microorganisms producing various enzymes such as cellulase, protease and phytase which help in breaking down of various complex food materials in its feed and thereby resulted in proper growth and development of the animal. Thapa et al. (2020) reported that the rumen of herbivorous animals including insects-dwelling microfloras are capable of hydrolyzing lignocellulose and other complex materials of which pullulans are included. The authors specifically observed rumen of herbivores as an effective biological system for degradation of complex macromolecules such as cellulose and pullulan, as the microbial communities residing the site synthesize various hydrolytic enzymes including pullulanase. Therefore, exploring mutualistic relationship between the herbivores such as worker termites and their microbiotas which have been found to be helpful in efficient and effective metabolism of complex materials, would unveil a hydrolytic enzyme such as pullulanase with industrial and biotechnological applications.

The identification of microorganisms was achieved using molecular techniques. The best three pullulolytic bacterial strains identified as *B. safensis*, *Psychrobacter pulmonis*, and *B. cereus*. Previous reports had it that pullulanases are extracellular enzyme and have been produced by different bacteria especially *Bacillus* species (Asha et al., 2013; Waleed et al., 2015). Molecular techniques gave more precision and accuracy more than conventional methods (Akinyemi and Oyelakin, 2014). The molecular identity of *Bacillus subtilis* producing pullulanase (Ogbo and Nwozor, 2020) was confirmed by i6s rRNA while Naik et al. (2021) identified endophytic fungi-producing pullulanase using ITS sequence analysis.

*Bacillus safensis*, *Psychrobacter pulmonis*, and *B. cereus* were further screened for pullulanase production at different incubation hours; whereas *B. safensis* had the highest pullulanase production after 12 h incubation period. The result obtained in this study is consistent with *Serratia marcescens* pullulanase (Femi-Ola et al., 2014). In contrast, *Bacillus* sp. and *Klibsiella pneumonia* were reported by Waleed et al. (2015) and Abdul-Hadi and Al-Bayyar (2019) to exhibit optimum pullulanase production after 48 h, while *Bacillus haloduran* (Asha et al., 2013) displayed maximum pullulanase at a very much higher incubation period of 5 days. Bacterial species often exhibit optimal enzyme production between incubation periods of 24–48 h (Niyonzina, 2019). The pullulanase production curve showed that *B. safensis* enzyme production began from exponential phase which depicted the bacterial ability to acclimatize itself to a new medium followed by its immediate utilization of the substrate (pullulan) and its resultant release of the enzyme into the medium within a short incubation period before its drastic decrease as the incubation hour progressed. Genomic differences may account for the varying incubation period exhibited by different bacterial species for production of enzymes (Niyonzina, 2019). The short incubation period is beneficial to several industrial processes as it could prevent contaminants and hasten processing period.

Purified extracellular pullulanase from *B. safensis* gave 13% yield and 24-fold purification after the protein was loaded on gel-filtration chromatography. The result obtained in this study is in tandem with *B. acidopullulyticus* pullulanase (Lappalainen et al., 1991) with 38% yield and 3.8-fold purification, Rehman et al. (2018) reported 47.5% yield and 13.6-fold purification for *Pyroculum calidifontis* recombinant type II pullulanase and white edible mushroom pullulanase (Shehata et al., 2016) had 20% recovery and 17.8-fold purification. However, Saha et al. (1988) achieved 42% yield, 3511- purification fold for *Clostridium thermohydrosulfuricum* pullulanase, while Kim et al. (1993) reported 11.3% yield and 101.6-fold purification for *Bacillus* sp. S-1. Abdul-Hadi Al-Bayyar (2019) gave account of 19% yield and 74.6-purification fold for *Klebsiella pneumonia* pullulanase. Adeseko et al., 2021, Adeseko et al., 2021a acknowledged that different purification stages and factors that protein are subjected to could be the cause of decrease in both protein purification fold and yield.

Using molecular technique, subunit molecular weight of the purified pullulanase (Pull) was estimated to be 42.34 kDa. The molecular mass obtained in this study is found to be lower than that of *Paenibacillus lautus*, 87.9 kDa (Chen et al., 2016). Much higher molecular weight was observed for pullulanase of *Klebsiella pneumonia* (94 kDa) (Abdul-Hadi and Al-Bayyar, 2019), *Bacillus acidopullulyticus* (102 kDa) (Lappalainen et al., 1991), *Pyrobaculum calidonfontis* (111 kDa) (Rehman et al., 2018), white edible mushroom (112 kDa) (Shehata et al., 2016), *Bacillus* sp (136 kDa) (Orhan et al., 2014). However, *Fervidobacterium pennavorans* exhibited exceptionally high native molecular weight of 240 kDa and was observed to contain three subunits having molecular mass of 77 kDa each (Koch et al., 1997). Adeseko et al. (2021b) reported determination of molecular weight of protein by SDS-PAGE to be sufficient but further studies on protein structural properties will require native state of the protein, hence, native gel electrophoresis would be mandated in this case.

The optimum pH 7.0 exhibited by *B. safensis* pullulanase isolated from the gut of termite is consistent with *Paenibacillus lautus* pullulanase (Chen et al., 2016), and *B. subtilis* and *Geobacillus thermoleovorans* NP 33 pullulanase (Nisha and Satyanarayana, 2018). Significantly, the enzyme exhibited high relative activity of 91 and 94, respectively at acidic pH 5.0 and 6.0 respectively. Various authors have reported microbes that exhibited different optimum pH for purified pullulanase, *C. thermohydrosulfuricum*, pH 5.0–5.5 (Saha et al., 1988), *B. acidopullulyticus*, pH 5.0 (Lappalainen et al., 1991), *Bacillus* sp. S-1, pH 6.0 (Kim et al., 1993) *K. pneumonia*, pH 6.0–7.0 (Abdul-Hadi and Al-Bayyar., 2019) and white edible mushroom, pH 9.0 (Shehata et al., 2016). The enzyme exhibited maximum stability at pH 5.0 with 80% residual activity after 3 h incubation, while significant residual activity of 58% was observed between pH 7–8. *C. thermohydrosulfuricum* (Saha et al., 1988) and *K. pneumoniae* (Abdul-Hadi and Al-Bayyar (2019)) pullulanase exhibited maximum stability at pH 3.0–5.0 and 6–7 respectively. Meanwhile, Chen et al. (2016) reported *Paenibacillus lautus* pullulanase to attain its pH stability at pH 6.5–9.0 with 80% residual activity. At extreme alkaline pH 9–11, *B. safensis* pullulanase reported in this study maintained about 40% residual activity over an incubation period of 3 h. Decreased optimum and stability of enzyme could be attributed to the shift in the enzyme active site due to ionization of the amino acids initiated by change in pH (Abdul-Hadi and Al-Bayyar (2019)). However, *B. safensis* pullulanase was stable over a wide range of pH in comparison with the previously reported works and therefore could serve several industrial processes.

The optimum temperature of 60 °C obtained in this study for *B. safensis* pullulanase is similar with *Bacillus* sp. S-1 (Kim et al., 1993) and *K. pnuemoniae* (Abdul-Hadi and Al-Bayyar (2019)) pullulanase. However, Chen et al. (2016) and Shehata et al. (2016) reported optimum 40 °C for *P. lautus* and edible mushroom respectively, Lappalainen et al. (1991), 50 °C for *B. acidopullulyticus*, while Saha et al. (1988) gave account of optimum temperature of 90 °C. *B. safensis* pullulanase showed to be thermally stable by exhibiting its maximum stability at 40 °C with 86% of its initial activity after 1 h incubation period, while it retained 76 and 66% residual activity at 50 and 60 °C respectively after 1 h. *K. pneumoniae* pullulanase retained 95% of its initial activity between 50 and 60 °C (Abdul-Hadi and Al-Bayyar (2019), Chen et al. (2016) reported pullulanase from *P. lautus* to be thermally stable at 40 °C following decreased enzyme stability at 45 and 50 °C after 60 and 40 min respectively, while *B. acidopullulyticus* was only stable at 50 °C and the activity was totally inactivated at 60 °C after 1 h (Lapplainen et al., 1991); meanwhile *C. thermohydrosulfuricum* exhibited maximum thermal stability at 90 °C (Saha et al., 1988). A thermostable amylolytic enzymes such as pullulanase are desirable in starch processing and other industrial process (Djekrif et al., 2018), therefore thermostability of *B. safensis* pullulanase could be exploited in such processes.

Enhancement of *B. safensis* pullulanase activity by Ca^2+^ was in consonant with *B. cereus* (Hii et al., 2009), *Paenibacillus lautus* (Chen et al., 2016), edible white mushroom (Shehata et al., 2016), *Pyrobaculum calidifontis* (Rehman et al., 2018) and *Clavispora lusitaniae* ABS7 (Dakhmouche et al., 2021), while the inhibition of the activity of the enzyme by Al^3+^, Ca^2+^, Fe^2+^, Pb^2+^, Co^2+^, Cu^2+^, Mg^2+^ ions and inhibitor such as EDTA was similar with that of *Cryptococcus* sp. (Djekrif et al., 2018) which was inhibited by Cu^2+^, Hg^2+^, Zn^2+^, Ni^2+^, Mg^2+^ and pullulanase from white edible mushroom (Shehata et al., 2016) strongly inhibited by Hg^2+^, Ag^+^ and Co^2+^. Pullulanase has been observed to be Ca^2+^-dependent (Nisha and Satyanarayana, 2018), while Rehman et al. (2018) in contrast reported pullulanase from *P. califonditis* to be Ca^2+^-independent. However, urea at concentrations investigated stimulated the activity of the enzyme, while SDS only enhanced the enzymatic activity at 1 mM; meanwhile, metal ions such as EDTA lowered the activity of the enzyme. White edible mushroom was enhanced in the presence of EDTA and DTT while it was inhibited by NaF and mercaptoethanol (Shehata et al., 2016) whereas Zebardast et al. (2017) observed inhibition of *Cohnella* amylopullulanase by urea.

The *K*_*m*_ and *V*_*max*_ of 0.324 μmol/ml/min and 6.85 mg/ml observed for pullulanase from *B. safensis* isolated from the gut of termite in this study is similar to that of *B. cereus* reported by Hii et al. (2009) with *V*_*max*_ of 0.275 μmol/min/ml and edible mushroom (Shehata et al., 2016) with *K*_*m*_ and *V*_*max*_ of 0.27 mg/ml and 0.75 μmol/min/ml.

Pullulanase purified from *B. safensis* associated with termite's gut has exhibited exceptional physicochemical properties: thermo-tolerance and thermal stability, wide range of pH and ability to endure surfactants-characteristics required for such starch debranching enzyme. *B. safensis* pullulanase would be a good candidate for processes such as food, pharmaceutics and other industrial and biotechnological applications.

## 7 Conclusion

The gut of termite has been established to produce array of bacteria with inherent pullulan-degrading ability. Pullulanase produced by *B. safensis* had the highest enzyme activity, while the homogeneity of the purified was established by single band molecular of 42.44 kDa. The purified *B. safensis* pullulanase was optimum at pH 7 and 60 °C which depicts that it is thermotolerant while it was however observed to be pH- and thermo-stable. Significantly, the enzyme exhibited exceptional metal ions and inhibitor tolerance even at high concentration while it also showed high affinity for the substrate (pullulan). The exceptional properties of *B. safensis* pullulanase could serve useful purposes in various industrial processes.

## Declarations

### Author contribution statement

Oladipo Oladiti Olaniyi: Conceived and designed the experiments; Contributed the reagents, materials and analysis tools or data.

Afolayan Olalekan Damilare: Performed the experiment.

Olusola Tosin Lawal: Analyzed and interpreted data; Wrote the paper.

Festus Omotere Igbe: Contributed reagents, materials, analysis tools or data.

### Funding statement

This research did not receive any specific grant from funding agencies in the public, commercial, or not-for-profit sectors.

### Data availability statement

Data included in article/supp. material/referenced in article.

### Declaration of interest's statement

The authors declare no conflict of interest.

### Additional information

No additional information is available for this paper.

## Appendix A Supplementary data

Supplementary material.docx Supplementary material.docx
