# Supplementary material for: Properties of a neutral, thermally stable and surfactant-tolerant pullulanase from worker termite gut-dwelling Bacillus safensis as potential for industrial applications
Source: Heliyon. 2022 Sep 13;8(9):e10617. doi: 10.1016/j.heliyon.2022.e10617 (PMC9489966; doi:10.1016/j.heliyon.2022.e10617)
Supplement: Supplementary material.docx [file mmc1.docx]

4a. Electrophoregram (by sodium dodecyl sulfate polyacrylamide gel electrophoresis (SDS-PAGE)) showing one band molecular weight of purified pullulan of B. safensis isolated from the gut of soldier termite.


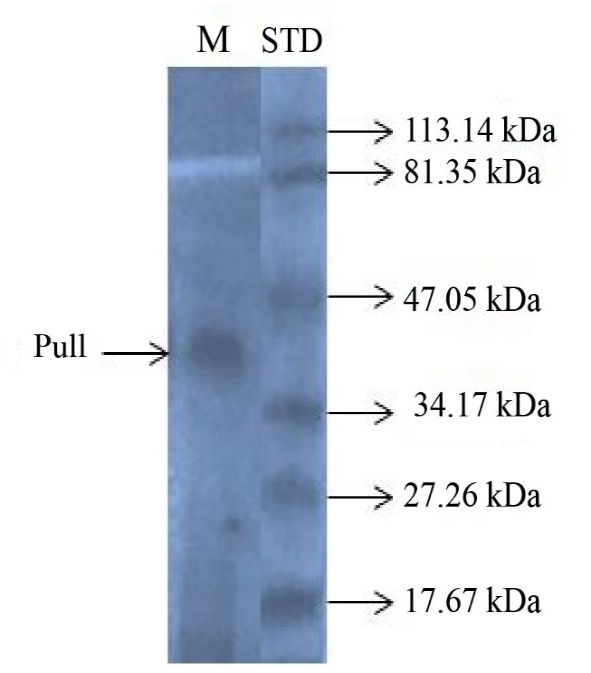


Figure 4a.

Lane M: purified pullulanase; Lane SD: Standard protein marker
